# Supplementary material for: Probing Electron Excitation Characters of Carboline-Based Bis-Tridentate Ir(III) Complexes
Source: Molecules. 2021 Oct 6;26(19):6048. doi: 10.3390/molecules26196048 (PMC8512491; doi:10.3390/molecules26196048)
Supplement: Supplementary file 1 [file molecules-26-06048-s001.zip › molecules-1399023-supplementary/Ir_carboline_SI_2021_09_29.pdf]

Supplementary Materials

Probing Electron Excitation Characters of Carboline Based Bis-tridentate Ir(III) Complexes

Jie Yan [1,‡], Ze-Lin Zhu [2,‡], Chun-Sing Lee [2,*], Shih-Hung Liu [3], Pi-Tai Chou [3,*] and Yun Chi [1,2,*]

[1] Department of Materials Science and Engineering, City University of Hong Kong, Hong Kong SAR, China; jyanae@connect.ust.hk (Y.J.)

[2] Department of Chemistry, Center of Super-Diamond and Advanced Films (COSDAF), City University of Hong Kong, Hong Kong SAR, China; zelinzhu-c@my.cityu.edu.hk (Z.-L.Z.); apcslee@cityu.edu.hk (C.-S.L.)

[3] Department of Chemistry, National Taiwan University, Taipei 10617, Taiwan. d96223131@ntu.edu.tw (S.-H.L.) chop@ntu.edu.tw (P.-T.C.)

‡ The authors who contributed equally.

* Correspondence: yunchi@cityu.edu.hk

Experimental Section

General information:

All solvents were dried and degassed before used, and commercially available reagents were used without further purification. 2,6-Dibromo-4-methoxypyridine,[1, 2] 2,6-dibromo-N,N-dimethylpyridin-4-amine[3, 4] and 6-(*tert*-butyl)-9H-pyrido[2,3-b]indole (^{tBu}cb)[5] were prepared using method in literature. All reactions were conducted under N_2 atmosphere and monitored by precoated TLC plates (0.20 nm with fluorescent indicator F254). ^{1}H and ^{19}F spectra were recorded with Bruker 400 MHz AVANCE III Nuclear Magnetic Resonance System. Elemental analysis was performed by an elemental Carbon-Hydrogen-Nitrogen analyzer (Elementar). Mass spectra were obtained on 4800 Plus MALDI TOF/TOF Analyzer (ABI), where 2,5-dihydroxybenzoic acid was applied as the matrix. TGA measurements were performed on a TA Instrument TGAQ50, at a heating rate of 10 °C min^{-1} under N_2 atmosphere. The X-ray intensity data were measured using phi and omega scans mode (APEX3) at 233 K on a Bruker D8 Venture Photon II diffractometer with microfocus X-ray sources.

Photophysical measurements:

All photophysical measurements in this study were performed at room temperature (298 K). UV-visible spectra were recorded on HITACHI UH4150. The emission spectra of the solution state were measured with an Edinburgh FL 980 fluorometer. Both wavelength-dependent excitation and emission responses of the fluorometer were calibrated. Steady-state absorption and emission spectra of the studied complexes were measured in dilute dichloromethane at room temperature, and coumarin 153 was used as standard, where spectral grade solvents were employed. To specify the quantum yield in the fluid state, samples were degassed using at least three freeze-pump-thaw cycles. The solution quantum yields are calculated using coumarin 153 that has a known quantum yield, according to the following equation:

$$\Phi = \Phi_R \frac{I}{I_R} \frac{A_R}{A} \frac{\eta^2}{\eta_R^2}$$

Where Φ is the PL quantum yield, the subscript R refers to the reference compound of known quantum yield, I is the integrated fluorescence intensity, and η is the refractive index of solvent. A is the absorbance at the excitation wavelength with the measured absorbance between 0.05 - 0.1.

Electrochemistry:

Cyclic voltammetry was conducted on a CHI660 Electrochemical Analyzer. Ag/Ag^+ (0.01 M $AgNO_3$) electrode was employed as the reference electrode. Oxidation and reduction potentials were measured using platinum working electrode with 0.1 M of NBu_4PF_6 as electrolyte in acetonitrile. The potentials were referenced externally to a ferrocenium/ferrocene (Fc^+/Fc) couple.

Device fabrication and measurement:

Pre-cleaned ITO coated glass substrates with a sheet resistance of 15 Ω $square^{-1}$ were subjected to UV-ozone treatment for 20 min. All the materials are purchased from Lumtec (Taiwan) and organic films were deposited at the rate of 0.5 ~ 1.0 Å s^{-1} by thermal evaporation in a deposition chamber with a base vacuum of 1×10^{-6} Torr. Current density–voltage (J-V) characteristics were recorded on a Keithley 237 power source (Tektronix). Luminance and electroluminescence spectra were measured by a PMA-12 photonic multichannel analyzer (Hamamatsu). Device measurements were performed under ambient conditions. The EQE was calculated according to the formula below:

$$EQE = \frac{\pi \cdot \mathrm{L} \cdot \mathrm{e}}{683 \cdot \mathrm{I} \cdot \mathrm{h} \cdot \mathrm{c}} \cdot \frac{\int_{380}^{780} I(\lambda) \cdot \lambda \mathrm{d}\lambda}{\int_{380}^{780} I(\lambda) \cdot \mathrm{K}(\lambda) d\lambda}$$

Where L (cd m^{-2}) is the total luminance of device, I (A) is the current flowing into the EL device, λ (nm) is EL wavelength, I(λ) is the relative EL intensity at each wavelength and

obtained by measuring the EL spectrum, K(λ) is the CIE chromaticity standard photopic efficiency function, e is the charge of an electron, h is the Planck's constant, c is the velocity of light.

Synthesis of the dianionic pro-chelates, (phyz1)H_2, (phyz2)H_2 and (phyz3)H_2.

Parent 2-phenyl-6-(3-(trifluoromethyl)-1H-pyrazol-5-yl)pyridine pro-chelate (phyz1)H_2 was synthesized from 2,6-dibromopyridine, using a multi-step protocol described in literature, c.f. Scheme S1.[6-8] The corresponding methoxy and dimethylamino substituted chelates, i.e. 4-methoxy-2-phenyl-6-(3-(trifluoromethyl)-1H-pyrazol-5-yl)pyridine (phyz2)H_2 and N,N-dimethyl-2-phenyl-6-(3-(trifluoromethyl)-1H-pyrazol-5-yl)pyridin-4-amine (phyz3)H_2, were also prepared using 2,6-dibromo-4-methoxypyridine and 2,6-dibromo-N,N-dimethylpyridin-4-amine as the starting materials, respectively.

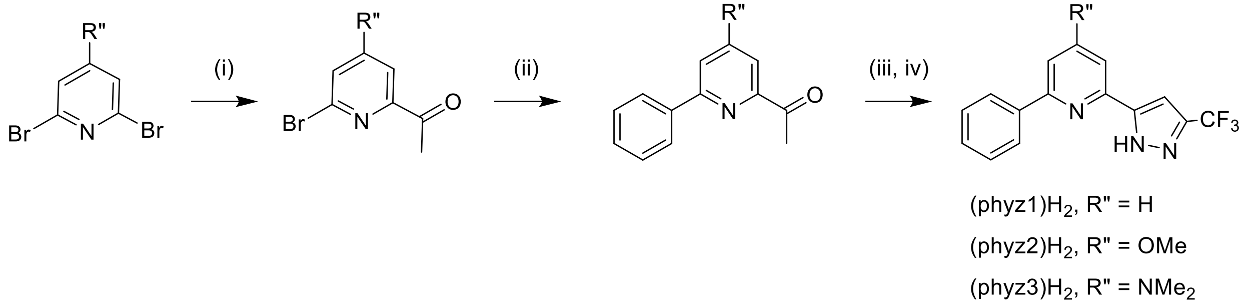

Scheme S1. Synthetic protocol given the employed dianionic chelates (phyz)H_2: experimental conditions: (i) *n*-BuLi, dimethylacetamide, dry ether, -78 °C to RT; (ii) phenyl boronic acid, Pd(PPh_3)$_2$$Cl_2$, K_2CO_3, reflux; (iii) NaOEt, ethyl trifluoroacetate, reflux; (iv) N_2H_4, *p*-toluenesulfonic acid, reflux.

Synthesis of the monoanionic carbene-benzene-carboline pro-chelates, (cbF)H·HF_6 and (cbB)H·HF_6.

These chelates were synthesized from 1,3-dibromo-5-(*tert*-butyl)benzene (and 1,3-

dibromo-5-(trifluoromethyl)benzene) and functional carboline using the multi-step protocol described in Scheme S2.

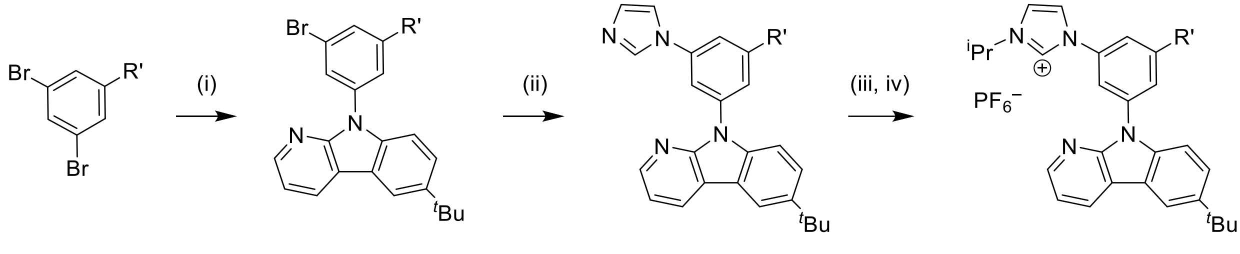

(cbF)H·HPF$_6$, R' = CF$_3$

(cbB)H·HPF$_6$, R' = ^{t}Bu

Scheme S2. Synthetic protocol given the employed carboline chelates (cbF)H·HF$_6$ and (cbB)H·HF$_6$: experimental conditions: (i) 6-(*tert*-butyl)-9H-pyrido[2,3-b]indole, CuI, K_3PO_4, *trans*-1,2-diaminocyclohexane, 1,4-dioxane, reflux; (ii) imidazole, CuO, K_2CO_3, DMSO, 150 °C; (iii) isopropyl iodide, acetonitrile, 110 °C; (iv) KPF_6, MeOH, RT.

Synthesis of 9-(3-bromo-5-(*tert*-butyl)phenyl)-6-(*tert*-butyl)-9H-pyrido[2,3-b]indole (XII)

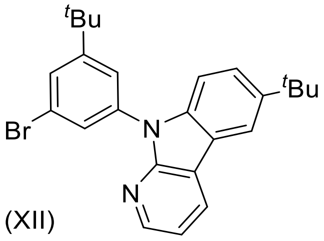

(XII)

A mixture of 1,3-dibromo-5-(*tert*-butyl)benzene (5.2 g, 17.8 mmol), **^{tBu}cb** (4.4 g, 19.6 mmol), CuI (1.0 g, 5.3 mmol), 1,2-diaminocyclohexane (1.0 g, 8.9 mmol), K_3PO_4 (11.3 g, 53.4 mmol) and 150 mL of dioxane was refluxed for 12 hours. After then, the solvent was evaporated, the residue was taken into ethyl acetate (80 mL × 2) and washed with deionized water. Finally, the organic layer was dried over Na_2SO_4, filtered, concentrated, and purified by column chromatography (SiO_2, EA/hexane = 1 : 9) to yield 4.6 g of yellow oil of **XII**, 3.1 mmol, yield 60 %.

Spectral data of **XII**: ^{1}H NMR (400 MHz, $CDCl_3$, 296 K): δ = 8.45 (dd, *J* = 5.2, 1.2 Hz, 1H), 8.38 (dd, *J* = 7.6, 1.2 Hz, 1H), 8.10 (d, *J* = 2.0 Hz, 1H), 7.63 (t, *J* = 2.0 Hz, 1H), 7.57 (d, *J*

= 1.6 Hz, 2H), 7.41 – 7.47 (m, 2H), 7.22 (d, *J* = 2.8 Hz, 1H), 1.44 (s, 9H), 1.35 (s, 9H).

Synthesis of (cbB)H·HF_6

A mixture of **XII** (3.7 g, 8.4 mmol), imidazole (0.72 g, 10.4 mmol), CuO (0.17 g, 2.1 mmol), and K_2CO_3 (2.9 g, 21 mmol) in 70 mL of degassed DMSO was heated at 150 °C for 24 hours. After then, DMSO was evaporated and the residue was taken into ethyl acetate (50 mL × 2) and washed with deionized water. The resulting organic layer was dried over Na_2SO_4, filtered, and concentrated, and purified by column chromatography (SiO_2, EA/hexane = 1 : 2) to yield 2.48 g of white solid. This white solid, together with isopropyl iodide (3.0 mL, 29.2 mmol), was transferred into a sealed tube charged with 40 mL of acetonitrile. The sealed tube was heated to 120 °C for 24 hours.

After then, the solvent was removed and, the residue was dissolved in 10 mL of methanol. Finally, 50 mL of concentrated KPF_6 aqueous solution was added to induce the immediate precipitation of a white solid. This mixture was continuously stirred at RT for 2 hours and the precipitate was collected and dried under vacuum to yield 3.5 g light yellow solid, 5.7 mmol, yield 68 %.

Spectral data of (cbB)H·HF_6: ^{1}H NMR (400 MHz, DMSO-d_6, 296 K): δ = 9.84 (s, 1H), 8.73 (dd, *J* = 7.6, 1.6 Hz, 1H), 8.42 – 8.48 (m, 2H), 8.36 (d, *J* = 1.6 Hz, 1H), 8.18 (s, 1H), 8.03 (s, 1H), 7.90 – 7.92 (m, 2H), 7.63 (dd, *J* = 8.8, 1.6 Hz, 1H), 7.50 (d, *J* = 8.8 Hz, 1H), 7.36 – 7.39 (m, 1H), 4.75 – 4.63 (m, 1H), 1.57 (d, *J* = 6.8 Hz, 6H), 1.44 (s, 9H), 1.43 (s, 9H); ^{19}F NMR (376 MHz, DMSO-d_6, 296 K): δ = -69.16 (d, J_{PF} = 710 Hz, 6F).

Synthesis of 9-(3-bromo-5-(trifluoromethyl)phenyl)-6-(*tert*-butyl)-9H-pyrido[2,3-b]indole (XIII)

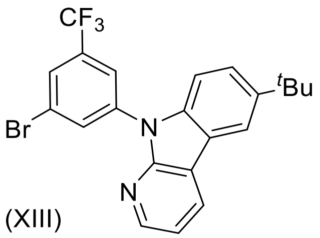

(XIII)

A mixture of 1,3-dibromo-5-(trifluoromethyl)benzene (873 mg, 2.9 mmol), ^{tBu}cb (0.5 g, 2.2 mmol), CuI (292 mg, 1.54 mmol), 1,10-phenanthroline (277 mg, 1.5 mmol) and K_2CO_3 (1.5 g, 11 mmol) in 25 mL of degassed 1,4-dioxane was refluxed for 12 hours. After then, the solvent was evaporated, the residue was extracted with ethyl acetate (50 mL × 2) and washed with deionized water. The organic layer was next dried over Na_2SO_4, filtered, concentrated, and purified by column chromatography (SiO_2, EA/hexane = 1 : 9) to yield 1.15 g of white solid, 2.3 mmol, yield 80 %.

Spectral data of XIII: ^{1}H NMR (400 MHz, $CDCl_3$, 296 K): δ = 8.52 (dd, *J* = 4.8, 1.6 Hz, 1H), 8.40 – 8.46 (m, 2H), 8.16 (d, *J* = 1.6 Hz, 1H), 8.11 (s, 1H), 7.82 (d, *J* = 8.8 Hz, 1H), 7.65 (dd, *J* = 8.8, 2.0 Hz, 1H), 7.29 – 7.32 (m, 2H), 1.49 (s, 9H); ^{19}F NMR (376 MHz, $CDCl_3$, 296 K): δ = -63.18 (s, 3F).

Synthesis of (cbF)H·HF_6

Following the same approach reported for (cbB)H·HF_6, (cbF)H·HF_6 was isolated as a yellow solid, yield 65%.

Spectral data of (cbF)H·HF_6: ^{1}H NMR (400 MHz, DMSO-d_6, 296 K): δ = 9.96 (s, 1H), 8.75 (dd, *J* = 7.8, 1.6 Hz, 1H), 8.59 (s, 1H), 8.52 (s, 1H), 8.47 (dd, *J* = 4.8, 1.5 Hz, 1H), 8.38 – 8.40 (m, 3H), 8.20 (t, *J* = 1.6 Hz, 1H), 7.61 – 7.67 (m, 2H), 7.41 – 7.44 (m, 1H), 4.69 – 4.76 (m, 1H), 1.58 (d, *J* = 6.8 Hz, 6H), 1.43 (s, 9H); ^{19}F NMR (376 MHz, DMSO-d_6, 296 K): δ = -63.18 (s, 3F), -71.92 (d, J_{PF} = 711 Hz, 6F).

Computational Method.

All calculations were performed with the Gaussian 16 program package.[9] The geometry optimization of ground states and excited states of Ir(III) complexes were simulated with density functional theory (DFT) at the hybrid functional PBE0/LANL2DZ (Ir) and PBE0/6-31g(d,p) (H, C, N, F, O) levels using CH_2Cl_2 as solvent. The single crystal X-ray structural data of **Cb1** has been used as the initial structural model for the geometrical optimization. The solvent effect is based on the polarizable continuum model (PCM), which is implemented in the Gaussian 16 program. Mulliken population analysis (MPA) was applied to obtain the electron density distribution of each atom in specific molecular orbital of Ir(III) complexes in order to calculate the metal-to-ligand charge transfer (MLCT) degree during each optical absorption and emission transition process.

Table S1. The calculated wavelengths, transition probabilities and charge transfer character of the optical transitions for Ir(III) complex **Cb1** in CH_2Cl_2.

State	λ (nm)	*f*	Assignments	MLCT
T_1	459.1	0	HOMO→LUMO+1(19%) HOMO-1→LUMO+1(18%) HOMO-1→LUMO+2(12%) HOMO-3→LUMO+2(11%)	13.52%
T_2	428.4	0	HOMO→LUMO+1(31%) HOMO→LUMO+2(13%) HOMO-3→LUMO+2(13%) HOMO-1→ LUMO+2(10%)	16.67%
T_3	426.6	0	HOMO→LUMO(72%) HOMO→LUMO+1(8%)	24.16%
S_1	402.7	0.0105	HOMO→LUMO+1(87%)	24.82%
S_2	389.1	0.0525	HOMO→LUMO(91%)	27.65%
S_3	366.4	0.0103	HOMO-1→LUMO+1(86%)	19.31%

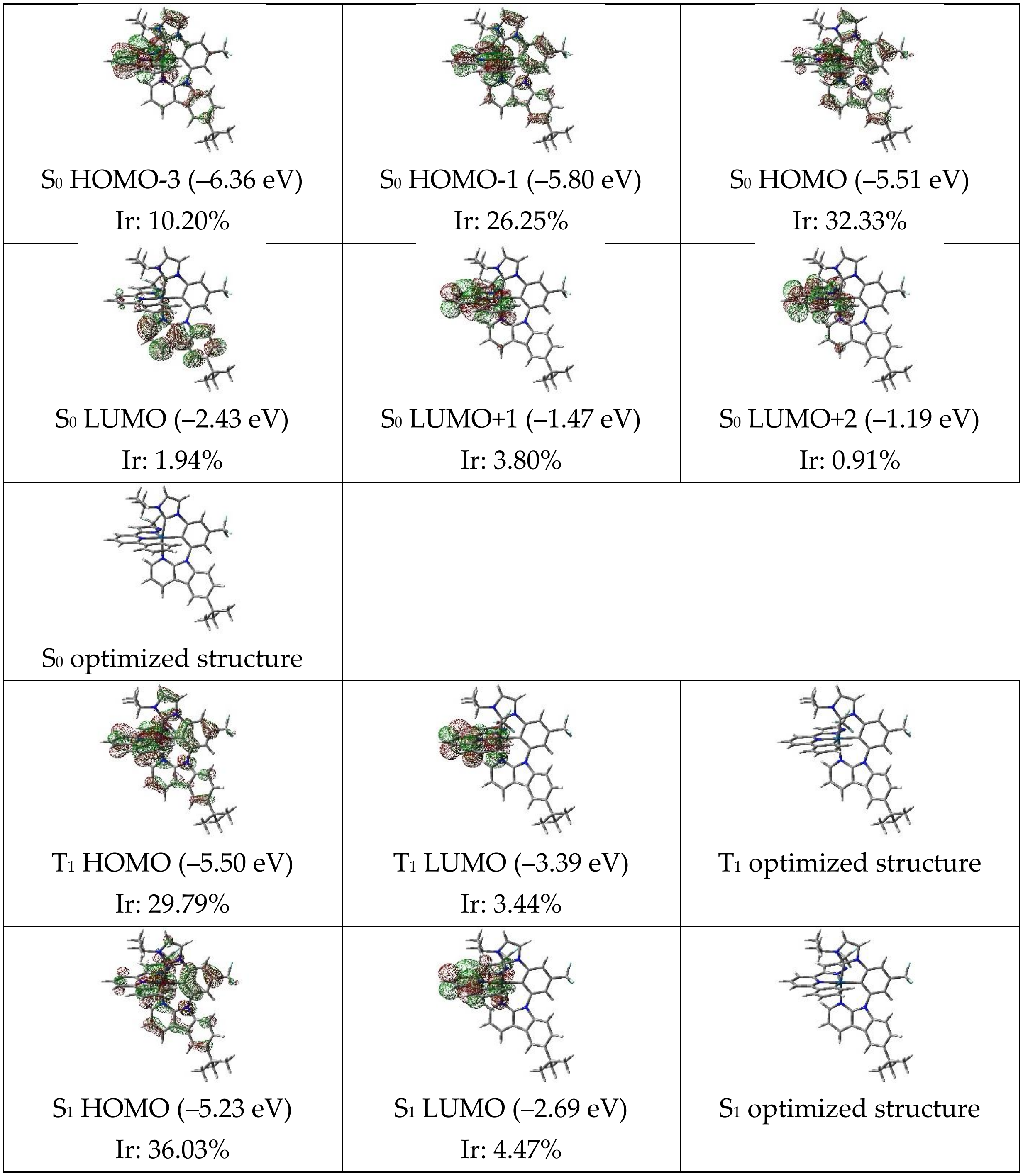

Figure S1. Frontier molecular orbitals pertinent to the optical transitions for the ground state S_0, excited state T_1 and S_1 of Ir(III) complex **Cb1**. The electron density distributions of Ir atom in each molecular orbital are showed.

Table S2. The calculated wavelengths, transition probabilities and charge transfer character of the optical transitions for Ir(III) complex **Cb2** in CH_2Cl_2.

State	λ (nm)	*f*	Assignments	MLCT
T_1	449.2	0	HOMO→LUMO(29%) HOMO→LUMO+1(29%) HOMO-3→LUMO+1(10%)	18.62%
T_2	428.2	0	HOMO→LUMO(79%)	24.52%
T_3	423.1	0	HOMO-5→LUMO(47%) HOMO-1→LUMO(6%)	1.77%
S_1	391.2	0.0425	HOMO→LUMO(94%)	29.18%
S_2	379.3	0.0364	HOMO→LUMO+1(84%)	26.10%
S_3	353	0.0062	HOMO-1→LUMO(58%) HOMO-1→LUMO+1(18%) HOMO→LUMO+2(10%)	22.74%

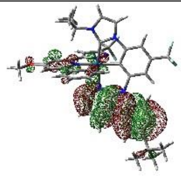<br>S_0 HOMO-5 (– 6.61 eV)<br>Ir: 2.38%	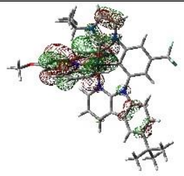<br>S_0 HOMO-3 (–6.35 eV)<br>Ir: 8.02%	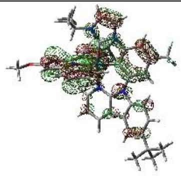<br>S_0 HOMO-1 (–5.78 eV)<br>Ir: 27.84%
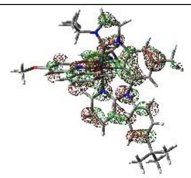<br>S_0 HOMO (–5.48 eV)<br>Ir: 32.96%	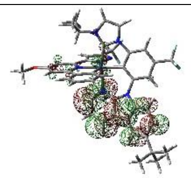<br>S_0 LUMO (–2.31 eV)<br>Ir: 1.92%	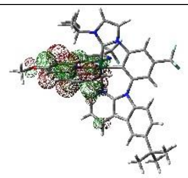<br>S_0 LUMO+1 (–1.32 eV)<br>Ir: 1.89%
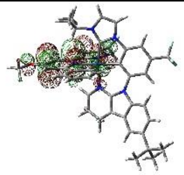<br>S_0 LUMO+2 (–1.06 eV)<br>Ir: 2.58%	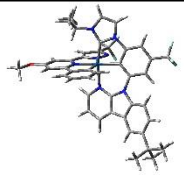<br>S_0 optimized structure	
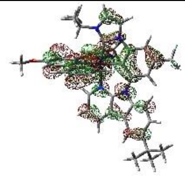<br>T_1 HOMO (–5.48 eV)<br>Ir: 30.79%	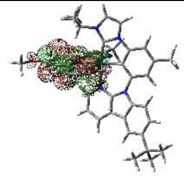<br>T_1 LUMO (–3.32 eV)<br>Ir: 2.85%	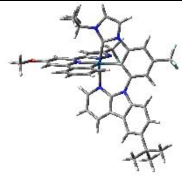<br>T_1 optimized structure
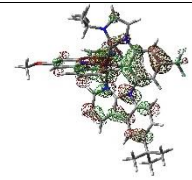<br>S_1 HOMO (–5.22 eV)<br>Ir: 32.90%	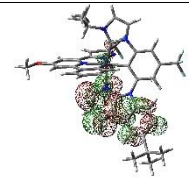<br>S_1 LUMO (–2.66 eV)<br>Ir: 2.28%	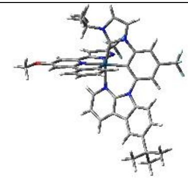<br>S_1 optimized structure

Figure S2. Frontier molecular orbitals pertinent to the optical transitions for the ground state S_0, excited state T_1 and S_1 of Ir(III) complex **Cb2**. The electron density distributions of Ir atom in each molecular orbital are showed.

Table S3. The calculated wavelengths, transition probabilities and charge transfer character of the optical transitions for Ir(III) complex **Cb3** in CH_2Cl_2.

State	λ (nm)	f	Assignments	MLCT
T_1	437.4	0	HOMO→LUMO(26%) HOMO-1→LUMO+1(22%) HOMO→LUMO+1(15%)	19.13%
T_2	430.5	0	HOMO→LUMO(58%) HOMO→LUMO+1(7%) HOMO-1→LUMO+1(7%)	22.87%
T_3	422.9	0	HOMO-5→LUMO(50%) HOMO-3→LUMO(7%) HOMO-1→LUMO(7%)	2.01%
S_1	394.8	0.0509	HOMO→LUMO(94%)	30.36%
S_2	361	0.0403	HOMO→LUMO+1(78%) HOMO→LUMO+2(14%)	29.79%
S_3	355.4	0.0088	HOMO-1→LUMO(94%)	24.62%

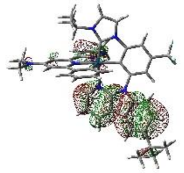<br>S_0 HOMO-5 (–6.56 eV)<br>Ir: 1.33%	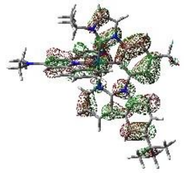<br>S_0 HOMO-3 (–6.21 eV)<br>Ir: 7.11%	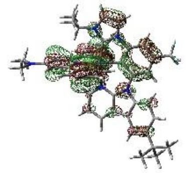<br>S_0 HOMO-1 (–5.70 eV)<br>Ir: 27.92%
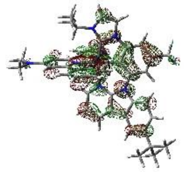<br>S_0 HOMO (–5.40 eV)<br>Ir: 34.03%	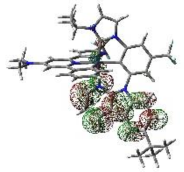<br>S_0 LUMO (–2.26 eV)<br>Ir: 1.73%	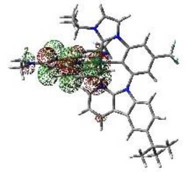<br>S_0 LUMO+1 (–1.09 eV)<br>Ir: 1.40%
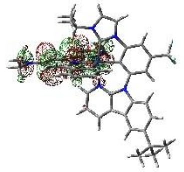<br>S_0 LUMO+2 (–0.88 eV)<br>Ir: 3.07%	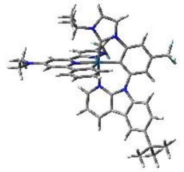<br>S_0 optimized structure	
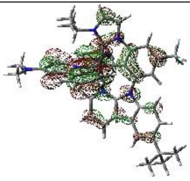<br>T_1 HOMO (–5.40 eV)<br>Ir: 30.52%	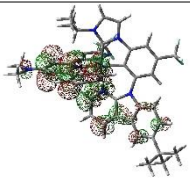<br>T_1 LUMO (–3.18 eV)<br>Ir: 2.86%	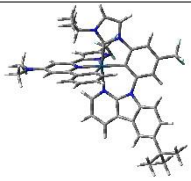<br>T_1 optimized structure
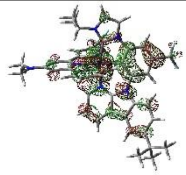<br>S_1 HOMO (–5.14 eV)<br>Ir: 34.22%	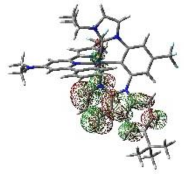<br>S_1 LUMO (–2.61 eV)<br>Ir: 2.31%	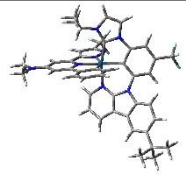<br>S_1 optimized structure

Figure S3. Frontier molecular orbitals pertinent to the optical transitions for the ground state S_0, excited state T_1 and S_1 of Ir(III) complex **Cb3**. The electron density distributions of Ir atom in each molecular orbital are showed.

Table S4. The calculated wavelengths, transition probabilities and charge transfer character of the optical transitions for Ir(III) complex **Cb4** in CH_2Cl_2.

State	λ (nm)	*f*	Assignments	MLCT
T_1	462.1	0	HOMO→LUMO+1(87%)	23.64%
T_2	442.2	0	HOMO→LUMO(91%)	26.40%
T_3	435.2	0	HOMO-1→LUMO+1(88%)	22.68%
S_1	417.8	0.0074	HOMO→LUMO+1(85%)	23.09%
S_2	406.9	0.0452	HOMO→LUMO(80%) HOMO-3→LUMO+2(11%)	23.99%
S_3	378.2	0.0101	HOMO-1→LUMO+1(83%)	21.39%

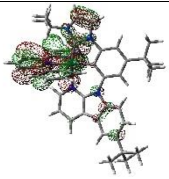<br>S_0 HOMO-3 (–6.28 eV)<br>Ir: 8.10%	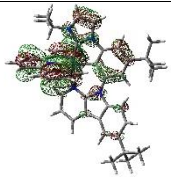<br>S_0 HOMO-1 (–5.66 eV)<br>Ir: 29.60%	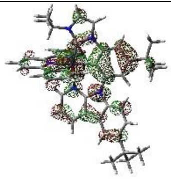<br>S_0 HOMO (–5.29 eV)<br>Ir: 31.00%
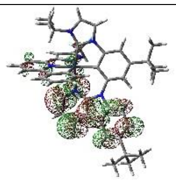<br>S_0 LUMO (–2.32 eV)<br>Ir: 1.99%	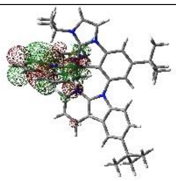<br>S_0 LUMO+1 (–1.40 eV)<br>Ir: 3.83%	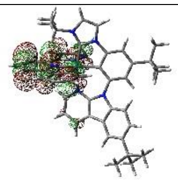<br>S_0 LUMO+2 (–1.13 eV)<br>Ir: 0.97%
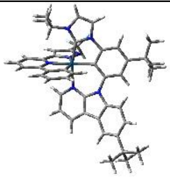<br>S_0 optimized structure		
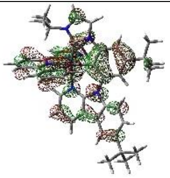<br>T_1 HOMO (–5.30 eV)<br>Ir: 30.14%	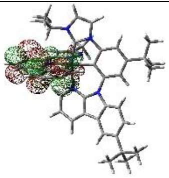<br>T_1 LUMO (–3.19 eV)<br>Ir: 3.54%	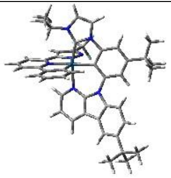<br>T_1 optimized structure
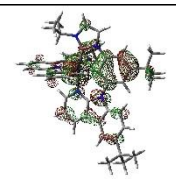<br>S_1 HOMO (–5.01 eV)<br>Ir: 32.90%	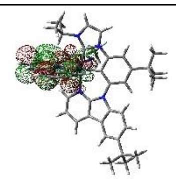<br>S_1 LUMO (–2.58 eV)<br>Ir: 4.37%	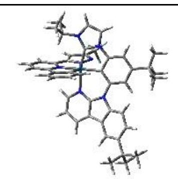<br>S_1 optimized structure

Figure S4. Frontier molecular orbitals pertinent to the optical transitions for the ground state S_0, excited state T_1 and S_1 of Ir(III) complex **Cb4**. The electron density distributions of Ir atom in each molecular orbital are showed.

Table S5. The calculated wavelengths, transition probabilities and charge transfer character of the optical transitions for Ir(III) complex **Cb5** in CH_2Cl_2.

State	λ (nm)	f	Assignments	MLCT
T_1	449.5	0	HOMO→LUMO(83%)	25.75%
T_2	437.2	0	HOMO-1→LUMO+1(34%) HOMO-3→ LUMO+2(27%)	10.18%
T_3	424.4	0	HOMO→LUMO+1(91%)	28.59%
S_1	413.4	0.0432	HOMO→LUMO(96%)	29.78%
S_2	375.3	0.03	HOMO→LUMO+1(86%) HOMO→LUMO+2(9%)	29.68%
S_3	361.1	0.0135	HOMO-1→LUMO(93%)	27.10%

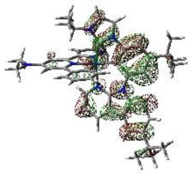<br>S_0 HOMO-3 (–5.94 eV)<br>Ir: 3.75%	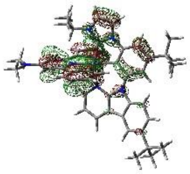<br>S_0 HOMO-1 (–5.56 eV)<br>Ir: 30.87%	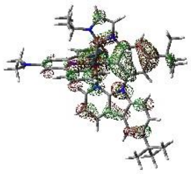<br>S_0 HOMO (–5.18 eV)<br>Ir: 32.75%
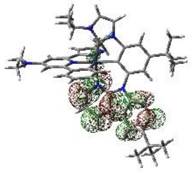<br>S_0 LUMO (–2.18 eV)<br>Ir: 1.73%	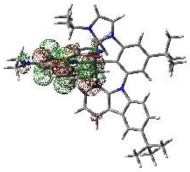<br>S_0 LUMO+1 (–1.04 eV)<br>Ir: 1.33%	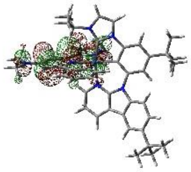<br>S_0 LUMO+2 (–0.82 eV)<br>Ir: 3.23%
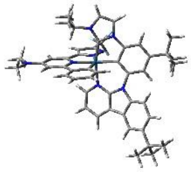<br>S_0 optimized structure		
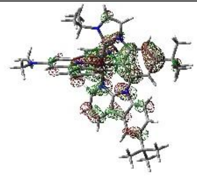<br>T_1 HOMO (–4.96 eV)<br>Ir: 34.09%	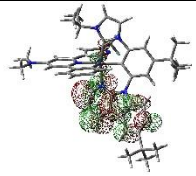<br>T_1 LUMO (–2.89 eV)<br>Ir: 2.71%	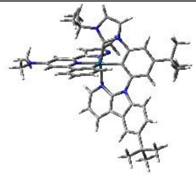<br>T_1 optimized structure
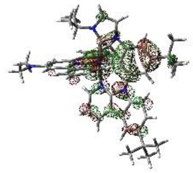<br>S_1 HOMO (–4.90 eV)<br>Ir: 31.73%	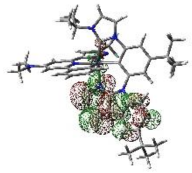<br>S_1 LUMO (–2.52 eV)<br>Ir: 2.28%	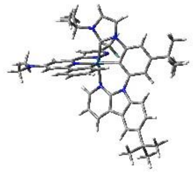<br>S_1 optimized structure

Figure S5. Frontier molecular orbitals pertinent to the optical transitions for the ground state S_0, excited state T_1 and S_1 of Ir(III) complex **Cb5**. The electron density distributions of Ir atom in each molecular orbital are showed.

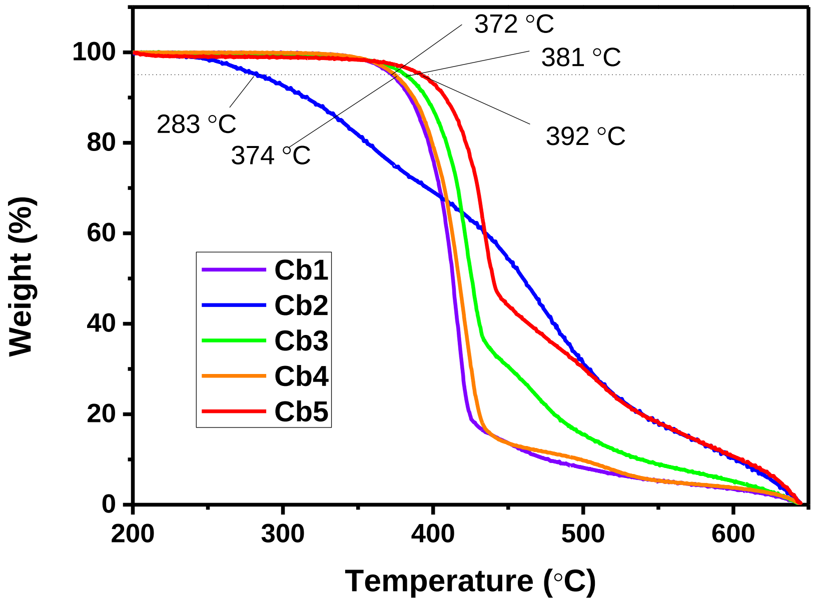

Figure S6. Thermal gravimetric analysis of studied Ir(III) complexes **Cb1** – **5** with decomposition temperature (T_d) showing a loss of 5% in weight.

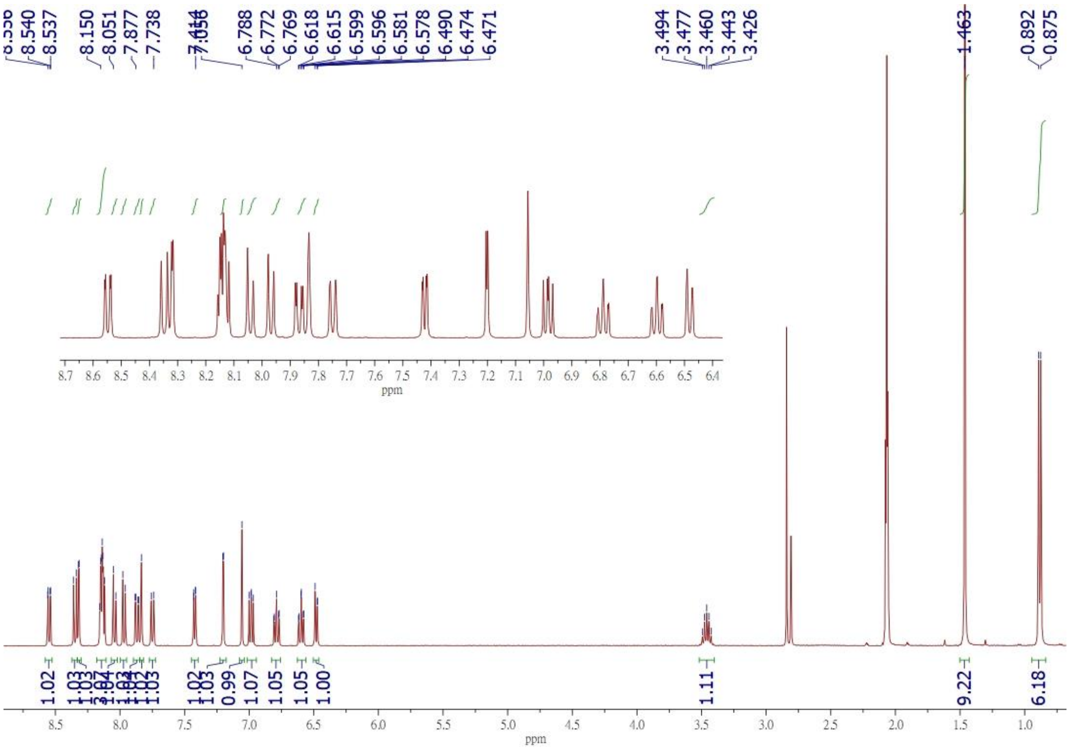

Figure S7. ^{1}H NMR spectrum of **Cb1** in acetone-d_6 at RT.

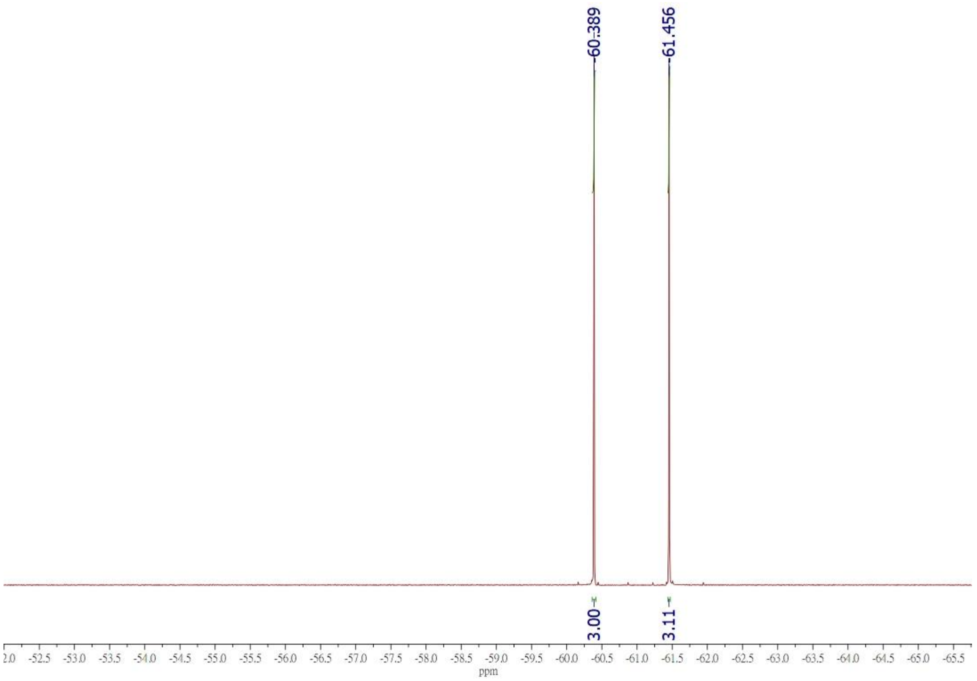

Figure S8. ^{19}F NMR spectrum of **Cb1** in acetone-d_6 at RT.

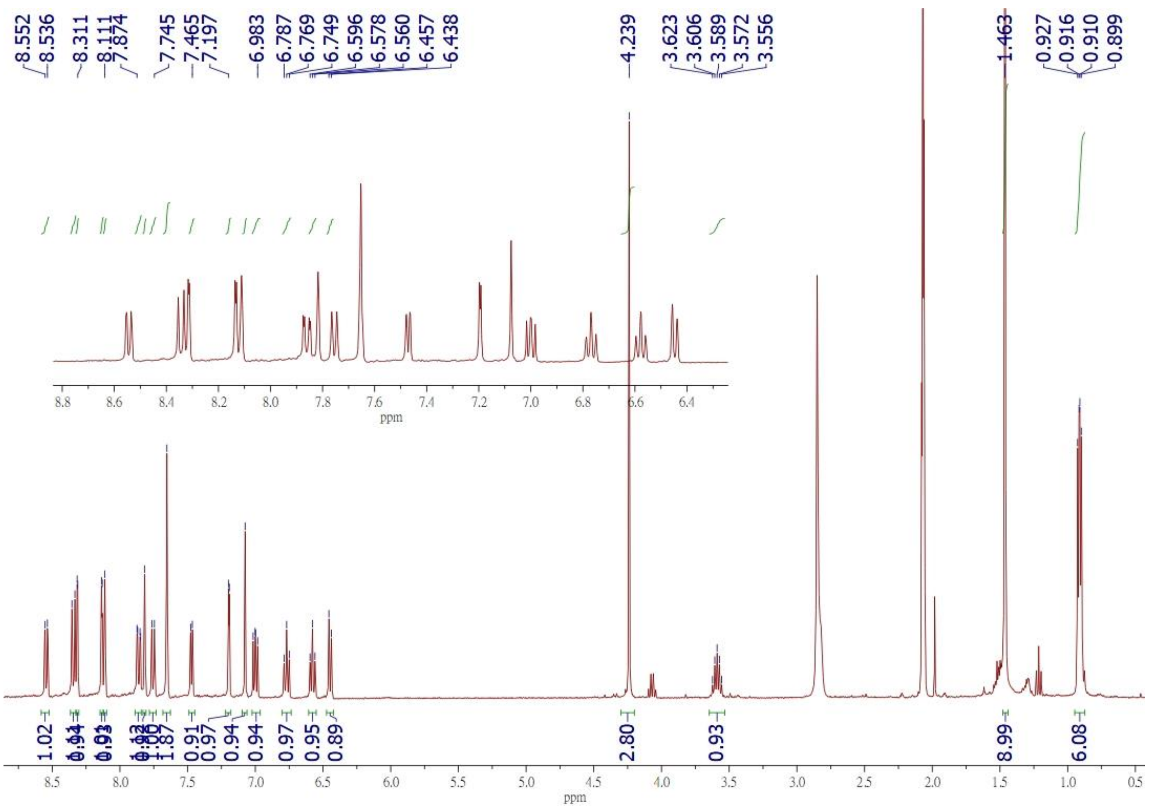

Figure S9. ^{1}H NMR spectrum of **Cb2** in acetone-d_6 at RT.

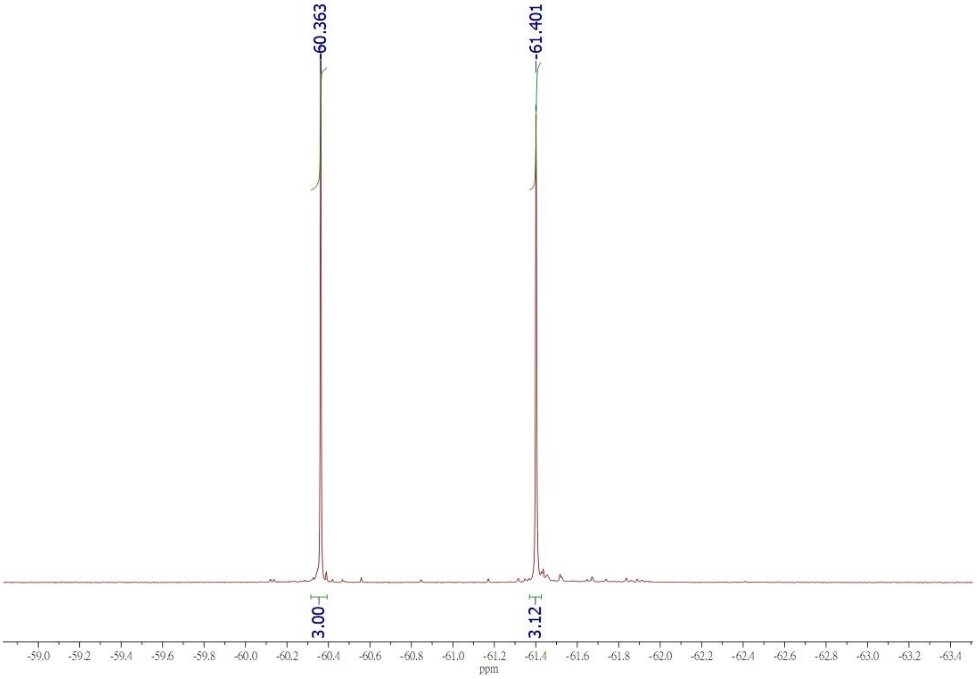

Figure S10. ^{19}F NMR spectrum of **Cb2** in acetone-d_6 at RT.

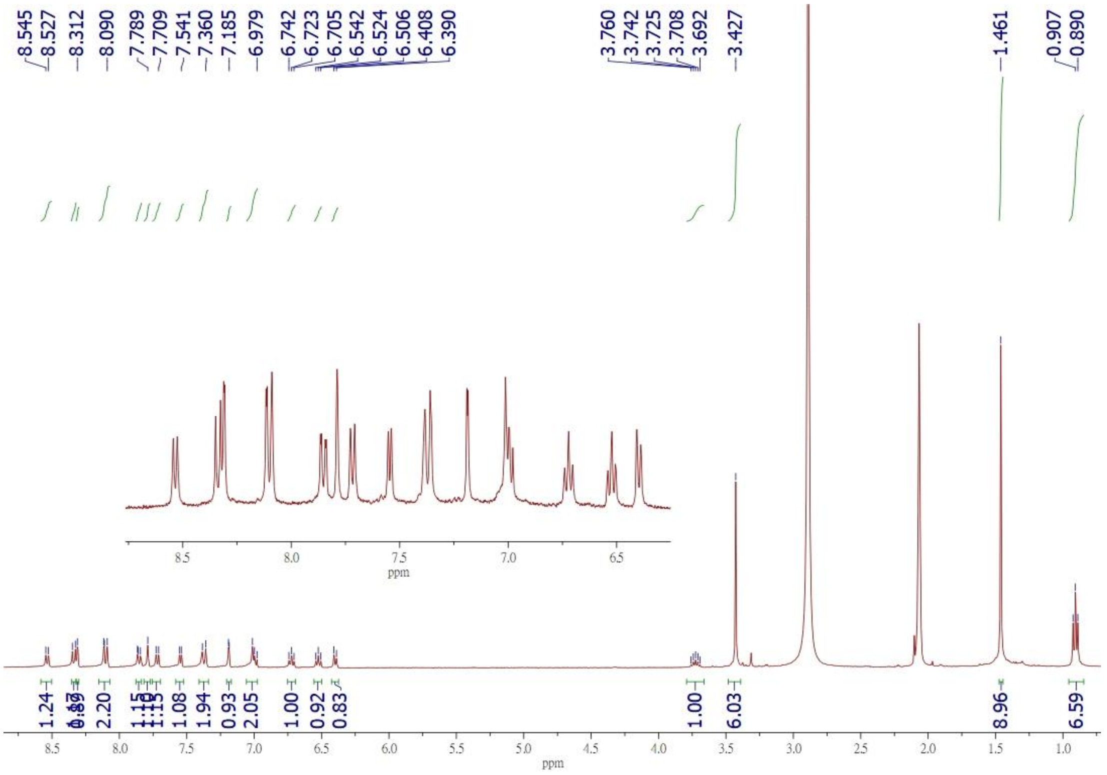

Figure S11. ^{1}H NMR spectrum of **Cb3** in acetone-d_6 at RT.

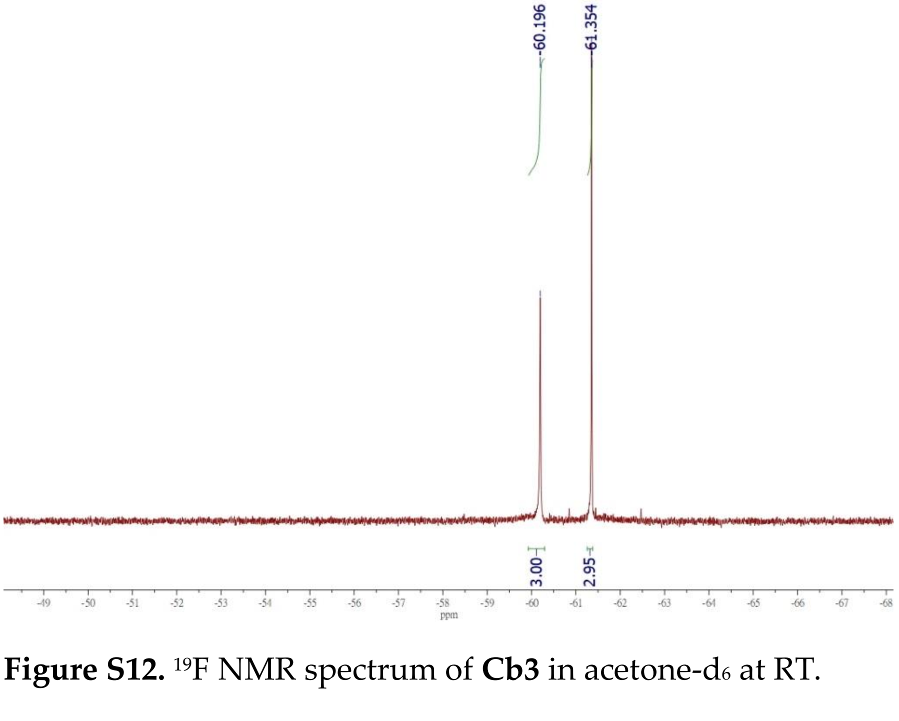

Figure S12. ^{19}F NMR spectrum of **Cb3** in acetone-d_6 at RT.

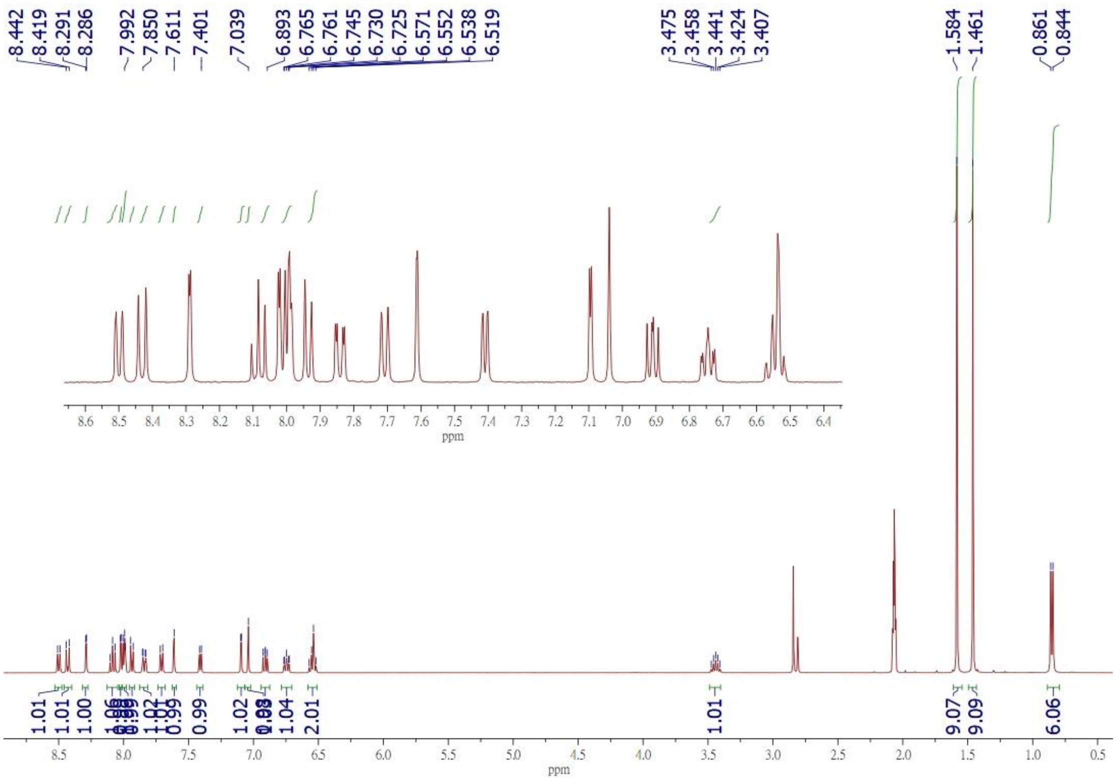

Figure S13. ^{1}H NMR spectrum of **Cb4** in acetone-d_6 at RT.

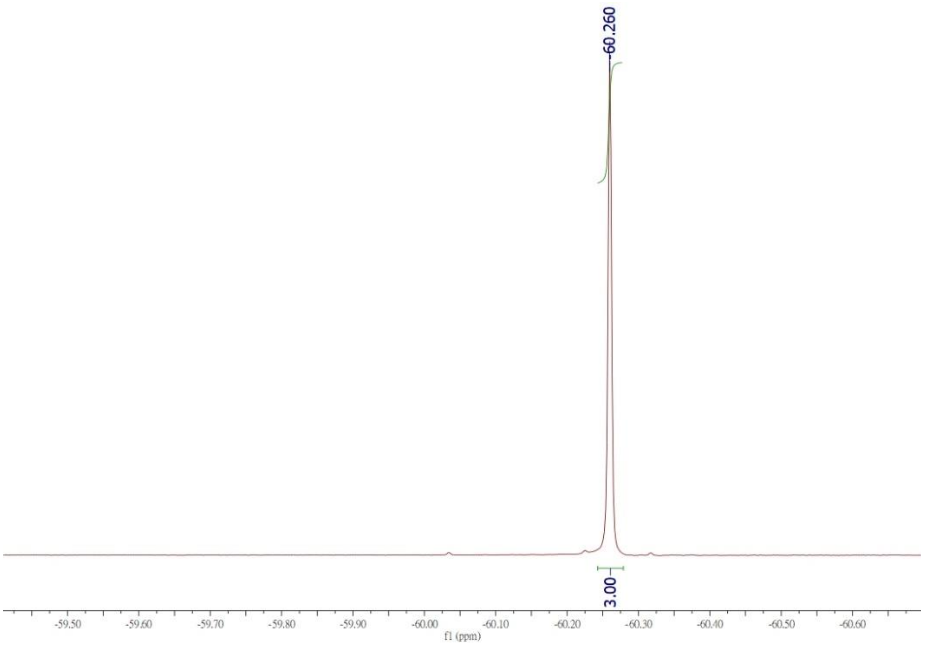

Figure S14. ^{19}F NMR spectrum of **Cb4** in acetone-d_6 at RT.

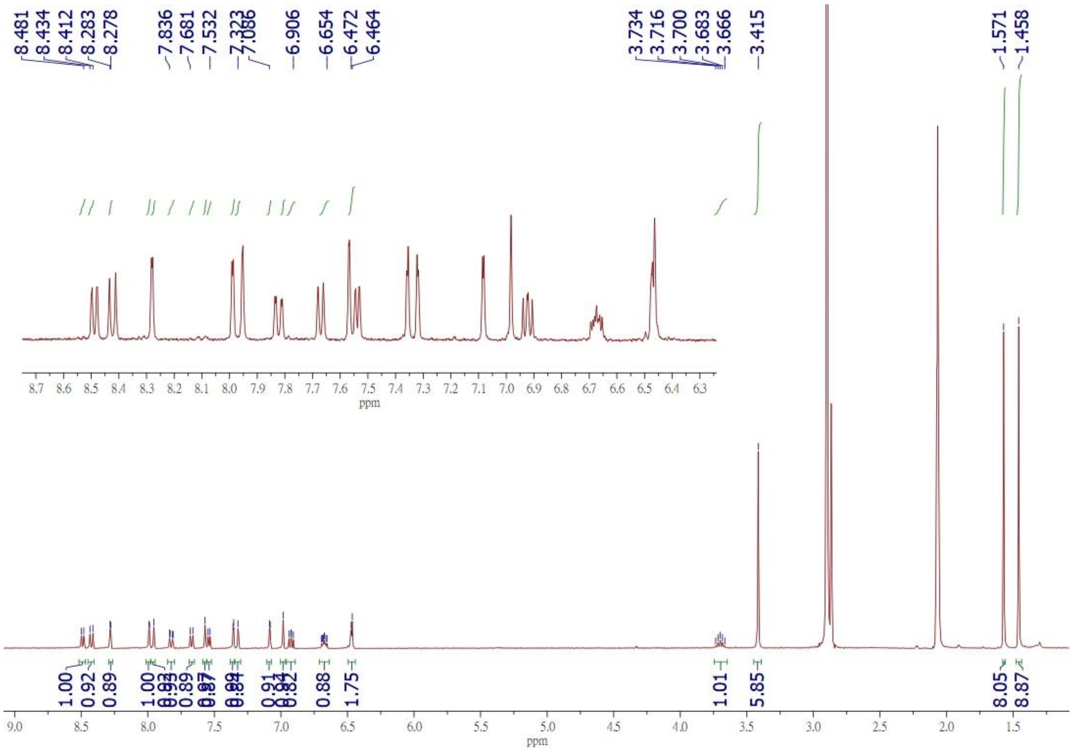

Figure S15. ^{1}H NMR spectrum of **Cb5** in acetone-d_6 at RT.

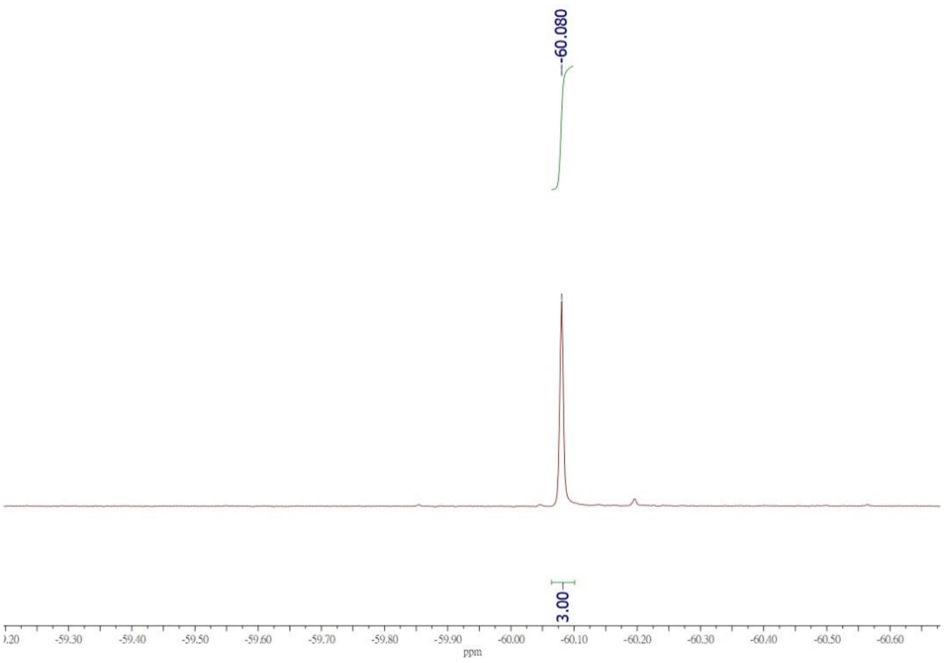

Figure S8. ^{19}F NMR spectrum of **Cb5** in acetone-d_6 at RT.

References

1. Neumann, U.; Vögtle, F., 4,4'-Donor-substituierte und 6,6'-difunktionalisierte 2,2'-Bipyridine. *Chem. Ber.* **1989,** 122, (3), 589-591.
2. Zhu, Z.-L.; Chen, W.-C.; Ni, S.-F.; Yan, J.; Wang, S. F.; Fu, L.-W.; Tsai, H.-Y.; Chi, Y.; Lee, C.-S., Constructing Deep-Blue Bis-tridentate Ir(III) Phosphors with Fluorene-Based Dianionic Chelates. *J. Mater. Chem. C* **2021,** 9, (4), 1318-1325.
3. Mairhofer, E.; Flemmich, L.; Kreutz, C.; Micura, R., Access to 3-Deazaguanosine Building Blocks for RNA Solid-Phase Synthesis Involving Hartwig–Buchwald C–N Cross-Coupling. *Org. Lett.* **2019,** 21, (11), 3900-3903.
4. Viricel, W.; Mbarek, A.; Leblond, J., Switchable Lipids: Conformational Change for Fast pH-Triggered Cytoplasmic Delivery. *Angew. Chem. Int. Ed.* **2015,** 54, (43), 12743-12747.
5. He, L.; Allwein, S. P.; Dugan, B. J.; Knouse, K. W.; Ott, G. R.; Zificsak, C. A., Synthesis of α-carboline. *Org. Synth.* **2016,** 93, 272-293.
6. Kuei, C.-Y.; Liu, S.-H.; Chou, P.-T.; Lee, G.-H.; Chi, Y., Room Temperature Blue Phosphorescence; A Combined Experimental and Theoretical Study on the Bis-tridentate Ir(III) Metal Complexes. *Dalton Trans.* **2016,** 45, (39), 15364-15373.
7. Kuei, C.-Y.; Tsai, W.-L.; Tong, B.; Jiao, M.; Lee, W.-K.; Chi, Y.; Wu, C.-C.; Liu, S.-H.; Lee, G.-H.; Chou, P.-T., Bis-Tridentate Ir(III) Complexes with Nearly Unitary RGB Phosphorescence and Organic Light-Emitting Diodes with External Quantum Efficiency Exceeding 31%. *Adv. Mater.* **2016,** 28, (14), 2795-2800.
8. Lin, J.; Wang, Y.; Gnanasekaran, P.; Chiang, Y.-C.; Yang, C.-C.; Chang, C.-H.; Liu, S.-H.; Lee, G.-H.; Chou, P.-T.; Chi, Y.; Liu, S.-W., Unprecedented Homoleptic Bis-Tridentate Iridium(III) Phosphors: Facile, Scaled-Up Production, and Superior Chemical Stability. *Adv. Funct. Mater.* **2017,** 27, (35), 1702856.
9. Frisch, M. J.; Trucks, G. W.; Schlegel, H. B.; Scuseria, G. E.; Robb, M. A.; Cheeseman, J. R.; Scalmani, G.; Barone, V.; Mennucci, B.; Petersson, G. A.; Nakatsuji, H.; Caricato, M.; Li, X.; Hratchian, H. P.; Izmaylov, A. F.; Bloino, J.; Zheng, G.; Sonnenberg, J. L.; Hada, M.; Ehara, M.; Toyota, K.; Fukuda, R.; Hasegawa, J.; Ishida, M.; Nakajima, T.; Honda, Y.; Kitao, O.; Nakai, H.; Vreven, T.; Montgomery, J. A.; Peralta, J. E.; Ogliaro, F.; Bearpark, M.; Heyd, J. J.; Brothers, E.; Kudin, K. N.; Staroverov, V. N.; Kobayashi, R.; Normand, J.; Raghavachari, K.; Rendell, A.; Burant, J. C.; Iyengar, S. S.; Tomasi, J.; Cossi, M.; Rega, N.; Millam, J. M.; Klene, M.; Knox, J. E.; Cross, J. B.; Bakken, V.; Adamo, C.; Jaramillo, J.; Gomperts, R.; Stratmann, R. E.; Yazyev, O.; Austin, A. J.; Cammi, R.; Pomelli, C.; Ochterski, J. W.; Martin, R. L.; Morokuma, K.; Zakrzewski, V. G.; Voth, G. A.; Salvador, P.; Dannenberg, J. J.; Dapprich, S.; Daniels, A. D.; Farkas, Ö.; Foresman, J. B.; Ortiz, J. V.; Cioslowski, J.; Fox, D. J., Gaussian 16, Revision A03. *Gaussian 16, Revision A03; Gaussian Inc.* **2016**, Wallingford, CT.
